# Electrical Stimulation Promotes Cardiac Differentiation of Human Induced Pluripotent Stem Cells

**DOI:** 10.1155/2016/1718041

**Published:** 2015-12-14

**Authors:** Damián Hernández, Rodney Millard, Priyadharshini Sivakumaran, Raymond C. B. Wong, Duncan E. Crombie, Alex W. Hewitt, Helena Liang, Sandy S. C. Hung, Alice Pébay, Robert K. Shepherd, Gregory J. Dusting, Shiang Y. Lim

**Affiliations:** ^1^O'Brien Institute Department, St Vincent's Institute of Medical Research, Fitzroy, VIC 3065, Australia; ^2^Department of Medicine, University of Melbourne, East Melbourne, VIC 3002, Australia; ^3^Bionics Institute, East Melbourne, VIC 3002, Australia; ^4^Medical Bionics Department, University of Melbourne, Parkville, VIC 3010, Australia; ^5^Centre for Eye Research Australia, Royal Victorian Eye and Ear Hospital, East Melbourne, VIC 3002, Australia; ^6^Department of Ophthalmology, University of Melbourne, East Melbourne, VIC 3002, Australia; ^7^School of Medicine, Menzies Institute for Medical Research, University of Tasmania, Hobart, TAS 7005, Australia; ^8^Department of Surgery, University of Melbourne, East Melbourne, VIC 3002, Australia

## Abstract

*Background.* Human induced pluripotent stem cells (iPSCs) are an attractive source of cardiomyocytes for cardiac repair and regeneration. In this study, we aim to determine whether acute electrical stimulation of human iPSCs can promote their differentiation to cardiomyocytes.* Methods*. Human iPSCs were differentiated to cardiac cells by forming embryoid bodies (EBs) for 5 days. EBs were then subjected to brief electrical stimulation and plated down for 14 days.* Results*. In iPS(Foreskin)-2 cell line, brief electrical stimulation at 65 mV/mm or 200 mV/mm for 5 min significantly increased the percentage of beating EBs present by day 14 after plating. Acute electrical stimulation also significantly increased the cardiac gene expression of* ACTC1*,* TNNT2*,* MYH7*, and* MYL7*. However, the cardiogenic effect of electrical stimulation was not reproducible in another iPS cell line, CERA007c6. Beating EBs from control and electrically stimulated groups expressed various cardiac-specific transcription factors and contractile muscle markers. Beating EBs were also shown to cycle calcium and were responsive to the chronotropic agents, isoproterenol and carbamylcholine, in a concentration-dependent manner.* Conclusions*. Our results demonstrate that brief electrical stimulation can promote cardiac differentiation of human iPS cells. The cardiogenic effect of brief electrical stimulation is dependent on the cell line used.

## 1. Background

After myocardial infarction, the heart fails to regenerate sufficiently to reestablish the normal cardiac function. The ability of stem cells to differentiate into cardiomyocytes for transplantation is a promising approach to repair and regenerate infarcted myocardium [1]. Specifically, human pluripotent stem cells such as embryonic stem cells (ESCs) and iPSCs are the most promising candidate cell types because of their ability to proliferate indefinitely in the undifferentiated state* in vitro* and to differentiate into* bona fide* contracting cardiomyocytes. However, application of ESCs and their derivatives have some limitations with anticipated immune rejection after transplantation. iPSCs, like ESCs, show pluripotency and self-renewal capabilities [2]. Patient-specific iPSCs, unlike ESC lines, offer an autologous source of stem cells and derivatives for personalized medicine, thus circumventing issues regarding immunological rejection [3, 4].

Spontaneous differentiation of human iPSCs into cardiomyocytes was first reported using the EB formation method [2, 5]. However, this method has a relatively low cardiac differentiation efficiency; a heterogeneous cell population is generated and it requires animal serum which limits their use in clinical application. Endogenous electric fields are present in the developing heart and are involved in cardiac development [6, 7]. Exogenous electrical stimulation has been applied to various cell types to promote cardiac differentiation including human fibroblasts [8], human mesenchymal stem cells [9, 10], human cardiac progenitor cells (c-kit/Sca-1) [11], mouse adipose-derived stem cells [12], and ESCs (both human and mouse) [13, 14]. Using electric fields that were within the endogenous physiological range (tens to hundreds of mV/mm) [7], it was found that electrical stimulation can direct the differentiation of the abovementioned cell types into immature cardiomyocyte-like cells. Here, we studied the effect of brief electrical stimulation on cardiac differentiation of human iPSCs.

## 2. Methods

### 2.1. Human iPSCs Generation

Human tissue sample collection was approved by the Human Research Ethics Committee of the Royal Victorian Eye and Ear Hospital (11/1031H). Following informed consent, CERA007c6 iPSCs were generated using skin fibroblasts from a 36-year-old healthy male by the episomal method as described previously [15, 16]. Briefly, passage 4 fibroblasts were nucleofected with episomal vectors expressing OCT4, SOX2, KLF4, L-MYC, LIN28, and shRNA against p53. The nucleofected fibroblasts were then replated onto mitotically inactivated mouse embryonic fibroblasts on day 7 and allowed to grow until day 34. Reprogrammed colonies resembling human embryonic stem cell morphology were manually dissected to establish clonal cell line for expansion. The established iPSC line was cultured on mitotically inactivated mouse embryonic fibroblasts feeders in the presence of DMEM/F-12 medium containing 1x GlutaMAX, 20% knockout serum replacement, 10 ng/mL basic fibroblast growth factor, 0.1 mM nonessential amino acids, 100 *μ*M *β*-mercaptoethanol, and 0.5x penicillin/streptomycin (all from Invitrogen, CA, USA). The iPS(Foreskin)-2 cell line generated from newborn fibroblasts by lentiviral transfection was obtained from Professor J. Thomson (University of Wisconsin) [4] and was used for all experiments unless otherwise indicated.

### 2.2. Cardiac Differentiation of Human iPSCs

Human iPSCs were maintained as previously described [17]. Cardiac differentiation of human iPSCs was induced through formation of EBs. EBs were formed by mechanically dissecting undifferentiated human iPSC colonies into approximately 0.2 mm^2^ pieces, transferred to low attachment plates, and cultured in suspension in serum-containing media (DMEM/F-12 GlutaMAX medium, 20% fetal bovine serum (Sigma-Aldrich, MO, USA), 0.1 mM 2-mercaptoethanol, 0.1 mM nonessential amino acids, and 50 U/mL penicillin/streptomycin, all from Invitrogen, unless otherwise stated) where they aggregated to form EBs over 5 days. On day 5 (day 0 after plating), EBs were transferred onto tissue culture plates precoated with 0.1% gelatin and 10 *μ*g/mL fibronectin (both from Sigma-Aldrich) and cultured in serum-free media containing RPMI medium (Invitrogen) supplemented with 284 nM ascorbic acid, 68.7 nM transferrin, 38.7 nM sodium selenite, and 0.0075% bovine serum albumin (all from Sigma-Aldrich). The percentage of contractile EBs was determined as the number of EBs that showed spontaneous contraction divided by the total number of EBs plated.

### 2.3. Electrical Stimulation

At day 5 of EB formation, EBs were subjected to electrical stimulation using a custom made electrical stimulator. The electrical stimulator consisted of 16 gold electrodes coated with platinum that fitted into an 8-well chamber slide (BD Falcon, MA, USA) (Figure 2(a)). Each well contained an anode and cathode with an interelectrode distance of 10 mm. The electrodes were connected to an electrical stimulator that generated a charge-balanced biphasic current pulses (Figure 2(b)). The EBs were then electrically stimulated in suspension (Figure 2(a)) for different durations (1.5, 5, 10, or 15 min). The electric fields studied were 65 and 200 mV/mm at 1 Hz frequency and 1 ms pulse width. Control EBs were subjected to the same procedure but without electrical stimulation. After electrical stimulation, EBs were immediately transferred onto gelatin and fibronectin coated culture plates and cultured in serum-free media.

### 2.4. Quantitative Polymerase Chain Reaction (RT-qPCR)

RNA was extracted from undifferentiated iPSC and beating EBs at day 14 after plating using TRI Reagent (Invitrogen) followed by RNA precipitation with chloroform and isopropanol (both from Sigma-Aldrich). In a separate experiment, RNA was extracted from a pool of 5 randomly selected EBs at days 0 (before electrical stimulation), 1, 3, and 7 after plating (without selecting beating EBs). cDNA was synthesized using the high-capacity cDNA reverse transcription kit (Applied Biosystems, CA, USA). A qRT-PCR was carried out using TaqMan Universal master mix, TaqMan gene expression assays, and the 7900HT Fast Real-Time PCR machine (all from Applied Biosystems) to assess the expression of 18s (Hs99999901_s1), GATA binding protein 4 (*GATA4*; Hs00171403_m1), NK2 homeobox 5 (*NKX2.5*; Hs00231763_m1), myocyte enhancer factor 2C (*MEF2C*; Hs00231149_m1), T-box 5 (*TBX5*; Hs00361155_m1), cardiac *α*-actin (*ACTC1*; Hs01109515_m1), cardiac troponin T (*TNNT2*; Hs01109515_m1), myosin light chain atria isoform 7 (*MYL7*; Hs01085598_g1), myosin light chain ventricular isoform 2 (*MYL2*; Hs00166405_m1), myosin heavy chain (*MYH7*; Hs00165276_m1), muscarinic cholinergic receptor-2 (*CHM2*; Hs01085598_gl), *β*-adrenergic receptor-1 (*ADRB1*; Hs0233048_s1), and *β*-adrenergic receptor-2 (*ADRB2*; Hs002440532_s1). All readings were performed in duplicate. The fold change was calculated by applying the comparative CT method (ΔΔCT) whereby the mRNA expression levels were normalized against the level of the housekeeping gene 18s (ΔCt) with the level of candidate genes in EBs at day 0 (Figures 3(c), 3(d), 4(b), and 4(c)) or undifferentiated iPSC samples (Figures 5(b) and 5(c)) were used as the reference genes (ΔΔCt). RT-qPCR reaction of samples with reverse transcriptase omitted (−RT) was also conducted to check the absence of genomic DNA and the specificity of the primer probes.

### 2.5. Immunocytochemistry

The primary antibodies used to confirm pluripotency of CERA007c6 iPSCs were OCT4 (5 *μ*g/mL, Santa Cruz Biotechnology, TX, USA) and TRA1-60 (5 *μ*g/mL, Millipore), followed by the appropriate Alexa Fluor-488 secondary antibodies. For* in vitro* differentiation, CERA007c6 iPSCs were allowed to form EBs for 12 days and subsequently plated down on gelatinized plates to further differentiate for 15 days. Immunocytochemistry is performed using the primary antibodies against alpha-fetoprotein (10 *μ*g/mL AFP, Millipore), alpha smooth muscle actin (10 *μ*g/mL, SMA, R&D Systems, MN, USA), and Nestin (10 *μ*g/mL, Abcam), followed by the Alexa Fluor-488 secondary antibody (10 *μ*g/mL; Invitrogen).

For quantitative assessment of cardiomyocyte differentiation, beating colonies at 14 days after plating were trypsinized into single cell suspension with 0.25% trypsin-EDTA and spun onto coated glass slides (4 min at 900 rpm; Shandon Cytospin 4, Thermo Fisher Scientific, MA, USA). Cells were fixed and permeabilized with 100% ice-cold methanol for 10 min at 4°C. Cells were then incubated with a serum-free blocking solution (Thermo Fisher Scientific) for 10 min prior to incubation with cardiac troponin T antibody (2 *μ*g/mL, mouse monoclonal IgG; Abcam, MA, USA) at 4°C overnight followed by a secondary antibody, Alexa Fluor-488 goat anti-mouse IgG for 60 min at room temperature.

For qualitative analysis, beating colonies at 14-15 days after plating were treated with 10 *μ*M of Y-27632 (Millipore, MA, USA), a Rho-associated kinase (ROCK) inhibitor, for 1-2 hours at 37°C to minimise apoptosis [15]. Cells were then washed with phosphate buffer saline (PBS) and dissociated with TrypLE Select (Invitrogen) at 37°C for 5 min. Serum-containing media were then added to neutralize the enzymatic reaction of TrypLE. Mixture of single cells and clumps was centrifuged at 400 g for 5 min, resuspended in serum-containing media supplemented with 10 *μ*M of Y-27632, and seeded onto gelatin and fibronectin coated 8-well chamber slides. After 48 h of incubation at 37°C, the media were removed and the cells were fixed with 4% paraformaldehyde for 30 min at room temperature followed by Triton X-100 (0.1% in PBS) for 10 min. Cells were then incubated with a serum-free blocking solution for 10 min prior to incubation with antibodies against cardiac troponin T (4 *μ*g/mL, mouse monoclonal IgG, Abcam), sarcomeric *α*-actinin (25 *μ*g/mL, mouse monoclonal IgG, Sigma-Aldrich), myosin heavy chain (2 *μ*g/mL, mouse monoclonal IgG, Abcam), and connexin 43 (1.2 *μ*g/mL, rabbit polyclonal IgG, Abcam) at 4°C overnight followed by Alexa Fluor-488 goat anti-mouse IgG (10 *μ*g/mL, Invitrogen) or Cy3 fluorescence-conjugated goat anti-rabbit IgG (7.5 *μ*g/mL, Jackson ImmunoResearch Laboratories, PA, USA) for 60 min at room temperature.

All preparations were counterstained with DAPI (1 *μ*g/mL, Invitrogen) for nuclear staining and mounted with fluorescent mounting medium (Dako, Victoria, Australia). Images were taken using a fluorescence microscope (BX-61, Olympus, Tokyo, Japan). For quantification, at least 100 cells were counted from 10 random fields.

### 2.6. Calcium Imaging

Intracellular free calcium (Ca^2**+**^) imaging was performed in beating EBs collected at day 14 after plating using the fluorescent calcium indicator Fluo-4 AM (Invitrogen). Cells were incubated with 2 *μ*g/mL of Fluo-4 AM for 20 min in serum-containing media at 37°C. Gray scale images were captured (15 frames per second) on an inverted microscope (IX71, Olympus) with a fluorescence source (X-Cite 120Q; Lumen Dynamics, ON, Canada) and a digital camera (DP72; Olympus). The fluorescence signal was visualized with a 492 nm excitation beam (excitation filter BP492/18 with a xenon light source).

### 2.7. Microelectrode Array Recordings

The electrophysiological properties of the cardiomyocytes derived from iPSCs were assessed using a microelectrode array (MEA) recording system (Multichannel Systems, Reutlingen, Germany). Beating EBs at 14 days after plating were microdissected and transferred onto MEA plates coated with 0.1% gelatin and 10 *μ*g/mL fibronectin and cultured in serum-containing media. The responsiveness of cells to isoproterenol hydrochloride (1–1000 nM) and carbamylcholine (1–1000 nM) (all from Sigma-Aldrich) was tested 4 days later in Krebs-Ringer buffer (composition in mM: 125 NaCl, 5 KCl, 1 Na_2_HPO_4_, 1 MgSO_4_, 20 HEPES, 5.5 glucose, and 2 CaCl_2_; pH 7.4, at 37°C). Beating EBs from each MEA plate were treated with all drugs in random order, and cells were allowed to recover to their baseline contraction in fresh Krebs-Ringer buffer between drug treatments. Extracellular field potentials and beating rate were recorded at baseline and at 2 min after the addition of the drugs. Data were analyzed offline with MC Rack software, version 4.3.5 (Multichannel Systems) for beating rate, RR interval, and extracellular field potential duration (FPD). RR interval was defined as the time elapsed between two consecutive beats, and FPD was defined as the time interval between the initial deflection of the field potential and the maximal local T wave. To avoid the influence of beat frequency on FPD, FPD measurements were normalized (corrected field potential duration (cFPD)) with the Bazett correction formula: cFPD = FPD/√ (RR interval).

### 2.8. Statistical Analysis

Data are expressed as mean ± SEM. Statistical analysis was performed on Graphpad Prism software (version 6). The significance of the differences was evaluated using the unpaired Student *t*-test and one-way analysis of variance (ANOVA), followed by Bonferroni multiple-comparison* post hoc* analysis where appropriate. Values of *p* < 0.05 were considered statistically significant.

## 3. Results

### 3.1. Generation of the iPSC Line CERA007c6

**Figure 1:**
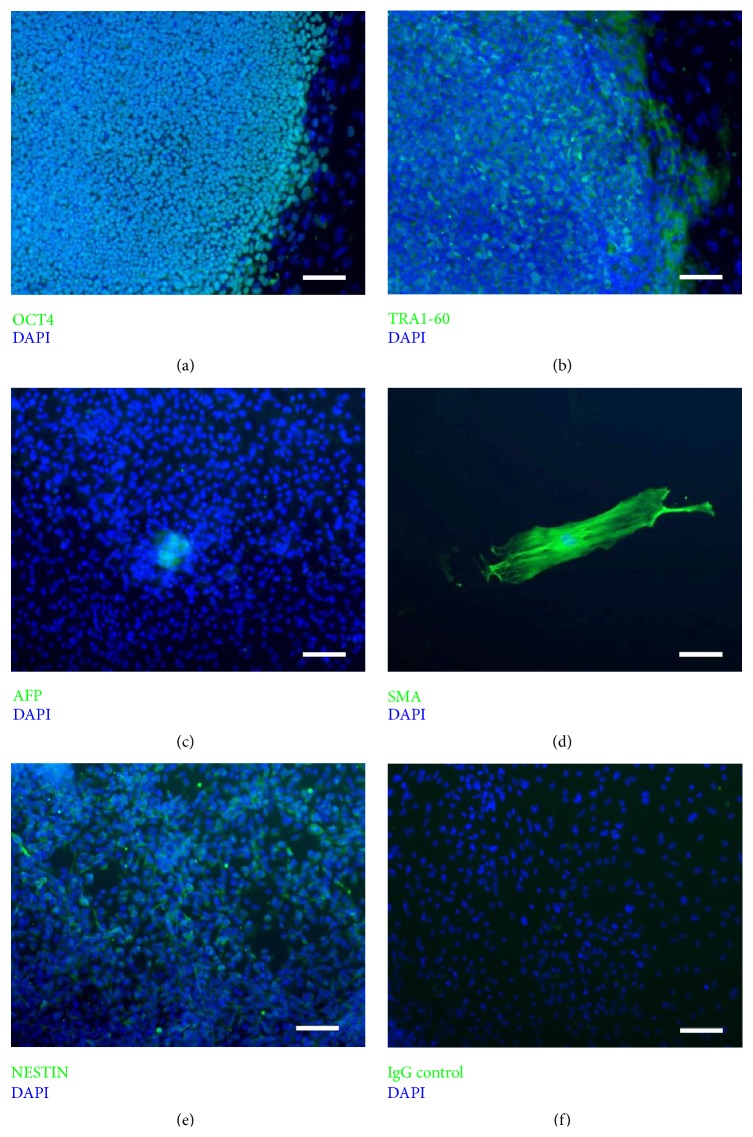
Characterisation of human iPSC line CERA007c6. Representative images showing expression of pluripotency markers (a) OCT4 and (b) TRA1-60 in the undifferentiated cells. CERA007c6 can be differentiated into all three lineages: (c) endoderm (alpha-fetoprotein, AFP), (d) mesoderm (smooth muscle actin, SMA), and ectoderm (Nestin). (f) An IgG negative control. Scale bar = 100 *μ*m.

**Figure 2:**
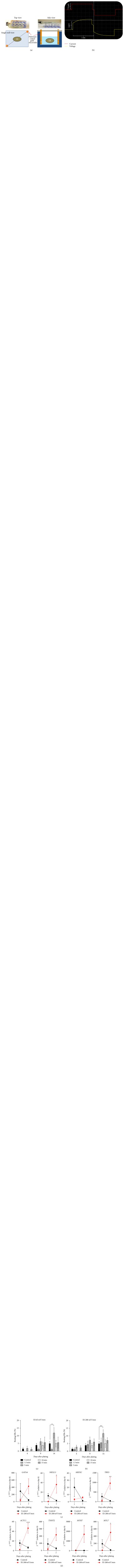
Custom made electrical stimulator. (a) A panel of 16 platinum-coated gold electrodes that fit into an 8-well chamber slide. Each well contained a pair of electrodes to stimulate the EBs in suspension. (b) Representative oscilloscope diagram of a pair of electrodes (at 200 mV/mm of the electric field) showing a charge-balanced biphasic current pulse (red) and the corresponding electrode voltage (yellow). Each current pulse was 1 ms/phase and was stimulated at 1 Hz. EB: embryoid body.

We generated new iPSC lines from skin fibroblasts of a healthy donor. We undertook nucleofection to deliver episomal vectors overexpressing OCT4, SOX2, KLF4, L-MYC, LIN28, and shRNA against p53 into fibroblasts. Following reprogramming, we isolated and expanded six iPSC clones. In the present study, clone 6 of the generated iPSCs was used (CERA007c6). Immunocytochemistry analysis showed that CERA007c6 iPSCs are positive for pluripotent markers OCT4 and TRA1-60 (Figures 1(a) and 1(b)). CERA007c6 iPSCs are differentiated* in vitro* by embryoid bodies formation, and we showed the presence of cells representative of endoderm (alpha-fetoprotein, AFP-positive cells), mesoderm (smooth muscle actin, SMA-positive cells), and ectoderm (Nestin-positive cells) (Figures 1(c)–1(e)). Together, these results confirmed that CERA007c6 iPSCs are pluripotent and retain the potential to differentiate* in vitro*. We have also characterised two other iPSC clones from the same donor and found consistent results (data not shown), confirming the quality of the iPSCs we generated.

### 3.2. Brief Electrical Stimulation Promotes Cardiac Differentiation of iPS(Foreskin)-2 Cell Line

**Figure 3:**
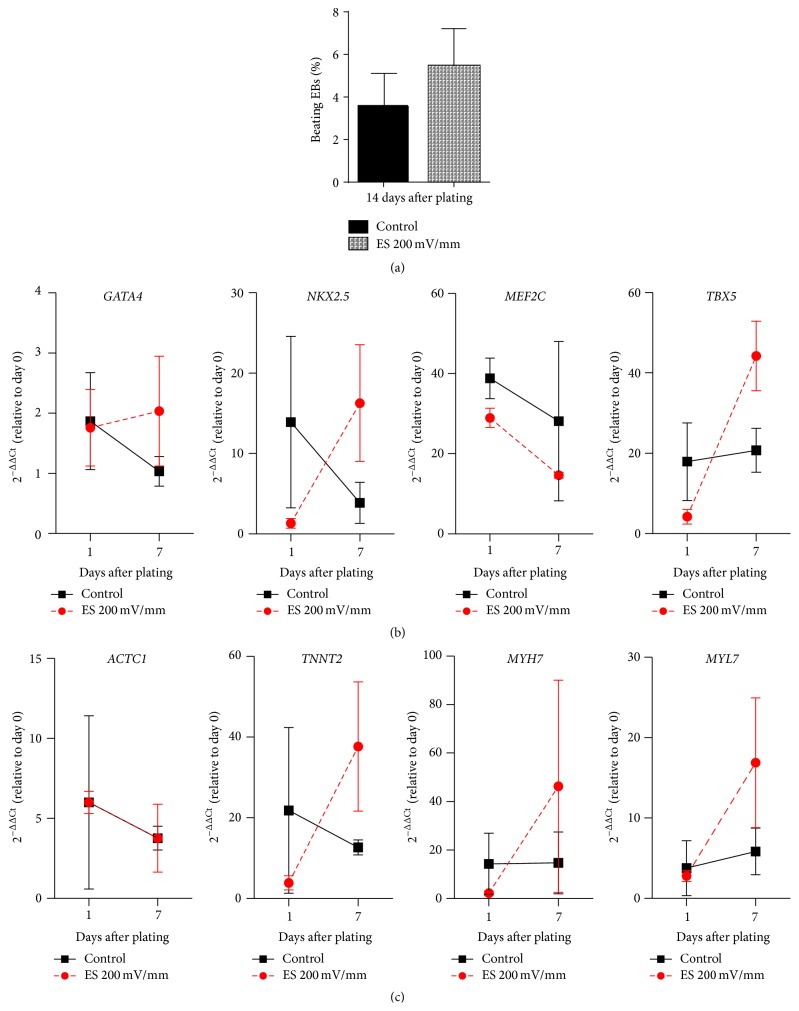
Acute electrical stimulation enhances cardiac differentiation of iPS(Foreskin)-2 cell line. (a-b) The effect of acute electrical stimulation at 65 mV/mm (a) or 200 mV/mm (b) on cardiac differentiation of human iPS(Foreskin)-2 cell line (*n* = 5–15 independent experiments). (c-d) Quantitative RT-PCR analysis for the expression of cardiac transcription factors (c) and cardiac contractile muscle proteins (d) in electrically stimulated and control EBs at days 0, 1, and 7 after electrical stimulation (*n* = 7 independent experiments). Data are expressed as mean ± SEM. ^*∗*^ *p* < 0.05, ^*∗∗*^ *p* < 0.01, and ^*∗∗∗*^ *p* < 0.001 by one-way (a-b) or two-way (c-d) ANOVA with the Bonferroni* post hoc* test.

Spontaneously beating colonies of iPS(Foreskin)-2 cells were detected as early as day 2 after plating but more frequently from day 4 after plating (Figures 3(a) and 3(b)). Acute electrical stimulation of EBs at 65 mV/mm (Figure 3(a)) or 200 mV/mm (Figure 3(b)) for 5 min statistically significantly increased the percentage of beating EBs at day 14 after plating (11.9 ± 3.4% in 65 mV/mm, 11.7 ± 2.3% in 200 mV/mm versus 4.9 ± 1.4% in control, one-way ANOVA, *p* < 0.05, *n* = 10–15). Electrical stimulation for a shorter or longer duration (1.5, 10, and 15 min) did not significantly modify the percentage of beating EBs when compared to control (Figures 3(a) and 3(b)). Electrical stimulation at either 65 or 200 mV/mm for a duration of 1.5 to 15 min did not affect the viability of EBs as determined by their gross morphology and electrically stimulated EBs were attached and grew after plating (data not shown). Compared to the unstimulated control group, acute electrical stimulation significantly increased the expression of cardiac transcription factors* NKX2.5* and* TBX5* (Figure 3(c)) as well as the cardiac contractile muscle proteins* ACTC1*,* TNNT2*,* MYH7*, and* MYL7* (Figure 3(d)) on day 7 after stimulation.

### 3.3. Brief Electrical Stimulation Did Not Promote Cardiac Differentiation of CERA007c6 Cell Line

**Figure 4:**
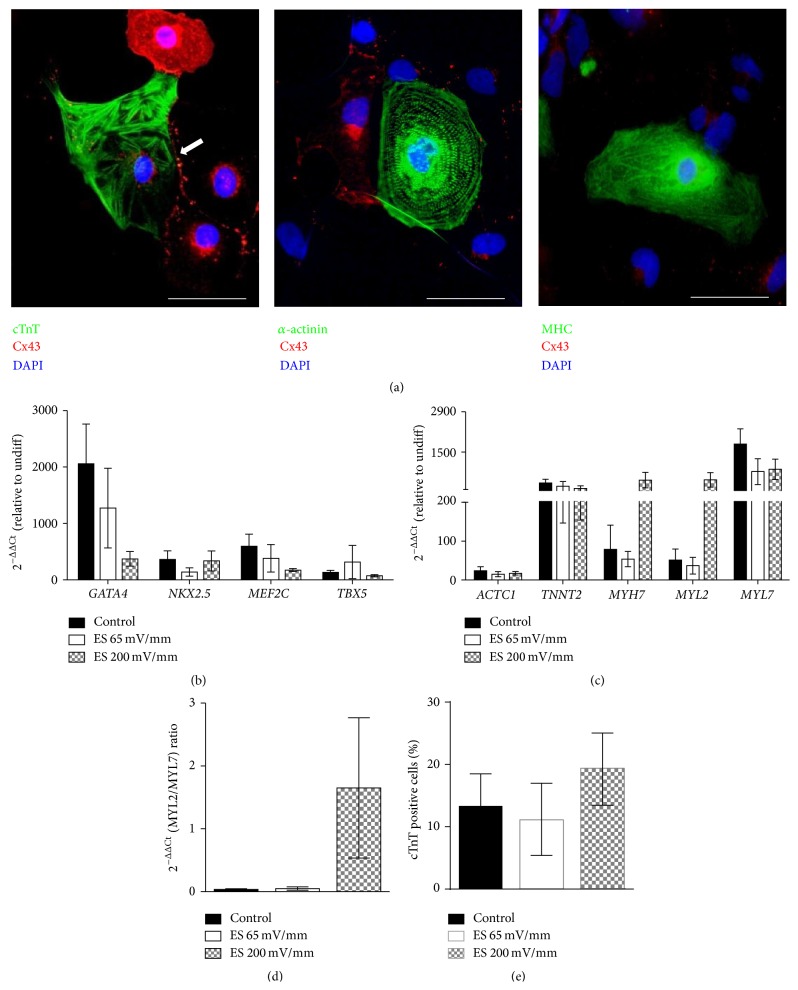
Acute electrical stimulation did not promote cardiac differentiation of CERA007c6 iPSCs. (a) The effect of acute electrical stimulation at 200 mV/mm for 5 min on cardiac differentiation of CERA007c6 iPSCs at day 14 after plating (*n* = 3 independent experiments). (b-c) Quantitative RT-PCR analysis for the expression of cardiac transcription factors (b) and cardiac contractile muscle proteins (c) in electrically stimulated and control EBs at days 0, 1, and 7 after electrical stimulation (*n* = 3 independent experiments). Data are expressed as mean ± SEM.

Acute electrical stimulation of EBs generated from the other line of human iPSC line, CERA007c6, at 200 mV/mm for 5 min did not significantly increase the percentage of beating EBs at day 14 after plating (5.5 ± 1.8% versus 3.6 ± 1.5% in control, *p* > 0.05, *n* = 8, Figure 4(a)). No significant difference in gene expression of cardiac transcription factors (Figure 4(b)) or cardiac contractile muscle proteins (Figure 4(c)) was detected between groups.

### 3.4. Expression of Cardiac-Specific Markers in Cardiomyocytes Derived from iPS(Foreskin)-2 Cell Line

**Figure 5:**
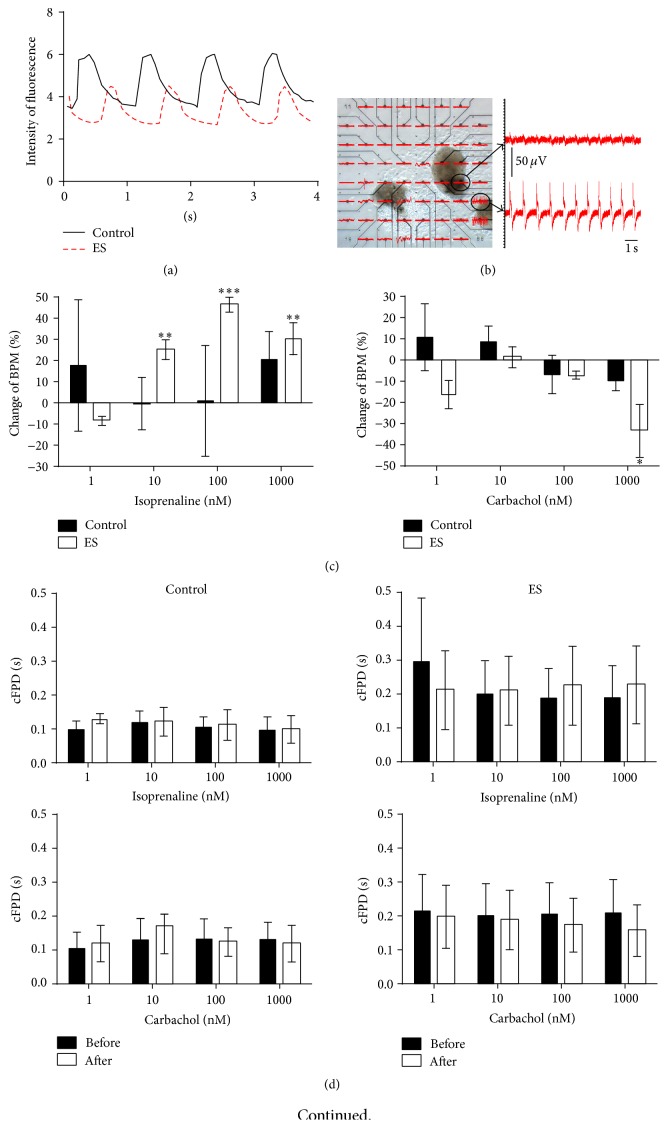
Expression of cardiac-specific markers in cardiomyocytes derived from iPS(Foreskin)-2 cell line. (a) Beating EBs expressed cardiac contractile proteins, cardiac troponin T, sarcomeric *α*-actinin, myosin heavy chain, and gap junctional protein connexin 43 (Cx43, white arrow) (scale bar = 50 *μ*m). Quantitative RT-PCR analysis for the expression of cardiac transcription factors (b) and cardiac contractile muscle proteins (c) in electrically stimulated and control beating EBs at day 14 after plating (*n* = 5–7 independent experiments). (d) MYL2/MYL7 gene expression ratio. (e) Percentage of cardiac troponin T-positive cells in each beating EB at day 14 after plating (*n* = 5-6 independent experiments). Data are expressed as mean ± SEM. ES: electrically stimulated and undiff: undifferentiated iPSCs.

At day 14 after plating, beating EBs expressed cardiac contractile proteins such as troponin T, sarcomeric *α*-actinin, and myosin heavy chain. Striations of cardiac muscle were visible in the cardiomyocytes derived from iPSCs (Figure 5(a)). In some cardiomyocytes, gap junctional protein connexin 43 was expressed in a punctuate expression pattern at the cell contact surfaces (Figure 5(a), white arrow) resembling that of neonatal cardiomyocytes [18].

Compared to undifferentiated human iPSCs, the mRNA expressions of cardiac transcription factors (*GATA4*,* NKX2.5*,* MEF2C,* and* TBX5*, Figure 5(b)) and cardiac contractile muscle proteins (*ACTC1*,* TNNT2*,* MYH7*,* MYL2, and MYL7*, Figure 5(c)) were upregulated in all beating EBs (both electrically stimulated and nonelectrically stimulated control). The gene expression ratio of* MYL2* (ventricular isoform)/*MYL7* (atrial isoform) was higher in beating EBs of the electrically stimulated group (at 200 mV/mm for 5 min) compared to control (1.70 ± 1.12 versus 0.03 ± 0.01, *p* < 0.05, *n* = 5–7, Figure 5(d)). Electrical stimulation at 65 mV/mm showed a similar gene expression ratio of* MYL2*/*MYL7* as did the control group (0.05 ± 0.03, *n* = 5, Figure 5(d)). These results suggest the emergence of more ventricular-like than atrial-like cardiomyocytes in beating EBs that were electrically stimulated at 200 mV/mm for 5 min.

The percentage of cardiac troponin T-positive cells in each beating EB from the iPS(Foreskin)-2 cell line was 13.4 ± 5.0% in control (*n* = 12). Acute electrical stimulation at 65 or 200 mV/mm for 5 min did not statistically increase the percentage of cardiac troponin T-positive cells (11.1 ± 5.9% and 19.4 ± 5.7%, resp., *p* > 0.05 versus control, one-way ANOVA, *n* = 6–12) (Figure 5(e)). In iPSC line CERA007c6, the percentage of cardiac troponin T-positive cells in each beating EB was 16.7 ± 10.0% in control (*n* = 5) and acute electrical stimulation at 200 mV/mm for 5 min did not significantly increase this percentage (30.5 ± 9.7%, *p* > 0.05 versus control, *n* = 6).

### 3.5. Electrophysiological Profile of Cardiomyocytes Derived from iPS(Foreskin)-2 Cell Line

**Figure 6:**
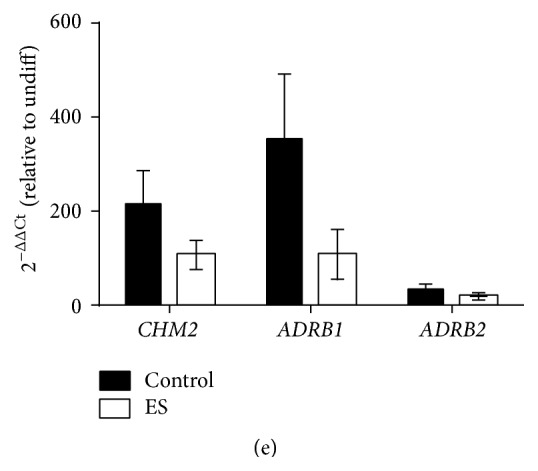
Electrophysiological properties of cardiomyocytes derived from iPS(Foreskin)-2 cell line. (a) Beating EBs at day 14 after plating were cycling calcium. Intensity of fluorescence has been normalized with background. (b) A representative image of a microelectrode array with beating pieces and the extracellular field potential recorded. (c) The percentage change in beating rate and (d) the cFPD of cardiomyocytes from control and electrically stimulated (ES, 200 mV/mm, 5 min) groups treated with isoproterenol hydrochloride (isoprenaline, 1–1000 nM) or carbamylcholine (carbachol, 1–1000 nM) (*n* = 3 independent experiments). (e) Quantitative RT-PCR analysis for the expression of muscarinic receptor 2 (*CHM2*), *β*-adrenergic receptor-1 (*ADRB1*), and *β*-adrenergic receptor-2 (*ADRB2*) in control and electrically stimulated beating EBs (*n* = 4–7 independent experiments). ES: electrically stimulated and undiff: undifferentiated iPSCs. Data are expressed as mean ± SEM. ^*∗*^ *p* < 0.05, ^*∗∗*^ *p* < 0.01, and ^*∗∗∗*^ *p* < 0.001 by one-way ANOVA with the Bonferroni* post hoc* test.

Beating cells from electrically stimulated and control beating EBs clearly cycle calcium (Figure 6(a)). To determine the electrophysiological properties of the beating EBs, extracellular electrograms were recorded from the beating clusters plated on top of MEA plates (Figure 6(b)). The resting beating rate per minute (bpm) was similar between control and electrically stimulated (200 mV/mm for 5 min) groups (60.7 ± 15.4 and 65.3 ± 12.7 bpm, resp., *p* > 0.05, *n* = 3). The beating EBs from the electrically stimulated group, but not the control group, showed a concentration-dependent increase in beating frequency when treated with isoproterenol (1–1000 nM, one-way ANOVA, *p* < 0.05, *n* = 3, Figure 6(c)). Treatment with 1000 *μ*M of carbamylcholine, a muscarinic agonist, significantly reduced the beating frequency in beating EBs from the electrically stimulated group, but not the control group (Figure 6(c)). Compared to control group, the baseline field potential duration (corresponding to the QT interval) trended higher in the electrically stimulated group but did not reach statistical significance (0.30 ± 0.19 sec versus 0.10 ± 0.02 sec in control, *p* > 0.05, *n* = 3, Figure 6(d)). The field potential duration remained unchanged when beating EBs were treated with either isoproterenol or carbamylcholine (Figure 6(d)). Thus beating EBs from the electrically stimulated group were more responsive to chronotropic agents but electrical stimulation did not significantly affect the field potential duration. Compared to undifferentiated human iPSCs, the mRNA expression of the muscarinic cholinoceptor (*CHRM2*) and *β*-adrenoceptors receptors (*ADRB1* and* ADRB2*) were upregulated in all beating EBs compared with undifferentiated cells but there was no significant difference between groups (Figure 6(e)).

## 4. Discussion

While studies with several cell types have shown that acute electrical stimulation can promote differentiation of cells toward cardiomyocyte phenotype [8–14], we have now shown that acute electrical stimulation increased cardiac differentiation of human iPSCs. In iPS(Foreskin)-2 cell line, acute electrical stimulation at 200 mV/mm for 5 min modestly but significantly increased the rate of cardiac differentiation of human iPSCs by twofold (Figure 3(b)). This was accompanied by a significant increase in expression of the cardiac-specific markers* ACTC1*,* TNNT2*,* MYH7, *and* MYL7*. Compared to previous studies in ESCs, we showed a positive effect of acute electrical stimulation on cardiac differentiation of human iPSCs at a much lower electric field (others used 500 to 1000 mV/mm) although we required longer stimulation duration (others required 1 to 90 seconds) [13, 14]. We were able to achieve differentiation at lower field strength than others perhaps due to our utilization of a constant current charge-balanced stimulation approach. It is surprising that acute electrical stimulation at 200 mV/mm did not promote cardiac differentiation using another human iPSC line (CERA007c6), and others have observed that human iPSC shows line to line variations and different differentiation efficacy [19]. This could be related to the cell origin from different patient (neonatal fibroblasts versus adult fibroblasts) or the reprograming method, which warrants further investigation.

Although acute electrical stimulation significantly increased the percentage of beating EBs, the percentage of cardiomyocytes determined by cardiac troponin T-positive cells in each beating EB was not significantly changed by acute electrical stimulation (Figure 5(e)). This variability in percentage of cardiomyocytes may be due to the variation in the size of the beating area and dilution by the variable number of noncardiomyocytes contained therein. The expression of cardiac progenitor transcription factors such as* GATA4*,* NKX2.5*,* MEF2C,* and* TBX5* is associated with early cardiac development and cardiomyocyte specification [20]. Once committed to the cardiac lineage, cells begin to express more mature cardiac markers such as those encoded for contractile muscle proteins* ACTC1*,* TNNT2*,* MYH7*,* MYL2,* and* MYL7* [21]. In the present study, the expression of these cardiac-specific transcription factors and structural genes was similar between the beating EBs from control and electrically stimulated groups, suggesting a comparable rate of cardiac specification and a similar proportion of cardiomyocytes in the beating EBs (Figures 5(b) and 5(c)).

Cardiomyocytes derived from human iPSCs showed development of calcium transients. Here we also showed that electrical stimulation increases the sensitivity of cardiomyocytes to isoproterenol and carbamylcholine when compared to the control group (Figure 6(c)). Fetal cardiomyocytes are less sensitive to isoproterenol than adult cardiomyocytes [22] suggesting the possibility that electrical stimulation may accelerate maturation of cardiomyocytes and thus explain the difference in sensitivity to *β*-adrenoceptor and muscarinic receptor agonists in the present study, although the expression of genes encoding these receptors was similar between the control and electrically stimulated beating EBs. The expression of *β*-adrenoceptor and muscarinic receptors at the protein level was not assessed, and the associated downstream signalling mechanism in the derived cardiomyocytes remains to be investigated. The lack of responsiveness to chronotropic agents of spontaneously differentiated cardiomyocytes in the control group was surprising but was in agreement with our previous finding using the same human iPSC line [17]. This could be due to the relatively “young” cardiomyocytes (day 18 after plating) used in the present study compared to others (usually >30 days) for long-term culture increases the maturity of cardiomyocytes in terms of calcium handling and the expression of ion channels, as well as cardiac structural and contractile proteins [23, 24].

The mechanism by which acute electrical stimulation promotes cardiac differentiation of human iPSCs remains unknown. Previous studies in mouse and human ESCs have implicated the involvement of reactive oxygen species and NF*κβ* signalling in the cardiogenic effect of acute electrical stimulation [13, 14]. There was a positive correlation between the increased cardiac differentiation rate and generation of reactive oxygen species intracellularly when ESCs were subjected to electrical stimulation. A similar increase in beating EBs was demonstrated when ESCs were treated with low concentration of hydrogen peroxide (1–10 nM) for one hour. Furthermore, the effect of acute electrical stimulation on the cardiac differentiation of these ESCs was abolished by free radical scavengers (dehydroascorbate and pyrrolidine dithiocarbamate) or the NF*κβ* antagonist (N-tosyl-L-phenylalanine chloromethyl ketone) [14].

## 5. Conclusions

We have shown here that a single brief period of electrical stimulation can promote cardiogenic potential of human iPSCs in terms of the number of beating EBs and gene expression of cardiac transcription factors and contractile muscle proteins. However, the cardiogenic effect of acute electrical stimulation was cell line dependent. The number of cardiomyocytes in each beating cluster was not significantly affected by acute electrical stimulation. The cardiomyocytes derived from electrically stimulated human iPSCs expressed cardiac-specific markers, cycled calcium, and were responsive to adrenergic and muscarinic stimuli.

## References

[1] Laflamme M. A., Murry C. E. (2011). Heart regeneration. *Nature*.

[2] Zhang J., Wilson G. F., Soerens A. G. (2009). Functional cardiomyocytes derived from human induced pluripotent stem cells. *Circulation Research*.

[3] Takahashi K., Yamanaka S. (2006). Induction of pluripotent stem cells from mouse embryonic and adult fibroblast cultures by defined factors. *Cell*.

[4] Yu J., Vodyanik M. A., Smuga-Otto K. (2007). Induced pluripotent stem cell lines derived from human somatic cells. *Science*.

[5] Zwi L., Caspi O., Arbel G. (2009). Cardiomyocyte differentiation of human induced pluripotent stem cells. *Circulation*.

[6] Jaffe L. F., Nuccitelli R. (1977). Electrical controls of development. *Annual Review of Biophysics and Bioengineering*.

[7] Nuccitelli R. (1992). Endogenous ionic currents and DC electric fields in multicellular animal tissues. *Bioelectromagnetics*.

[8] Genovese J. A., Spadaccio C., Langer J., Habe J., Jackson J., Patel A. N. (2008). Electrostimulation induces cardiomyocyte predifferentiation of fibroblasts. *Biochemical and Biophysical Research Communications*.

[9] Genovese J. A., Spadaccio C., Chachques E. (2009). Cardiac pre-differentiation of human mesenchymal stem cells by electrostimulation. *Frontiers in Bioscience*.

[10] Mooney E., Mackle J. N., Blond D. J.-P. (2012). The electrical stimulation of carbon nanotubes to provide a cardiomimetic cue to MSCs. *Biomaterials*.

[11] Pietronave S., Zamperone A., Oltolina F. (2014). Monophasic and biphasic electrical stimulation induces a precardiac differentiation in progenitor cells isolated from human heart. *Stem Cells and Development*.

[12] Pavesi A., Soncini M., Zamperone A. (2014). Electrical conditioning of adipose-derived stem cells in a multi-chamber culture platform. *Biotechnology and Bioengineering*.

[13] Serena E., Figallo E., Tandon N. (2009). Electrical stimulation of human embryonic stem cells: cardiac differentiation and the generation of reactive oxygen species. *Experimental Cell Research*.

[14] Sauer H., Rahimi G., Hescheler J., Wartenberg M. (1999). Effects of electrical fields on cardiomyocyte differentiation of embryonic stem cells. *Journal of Cellular Biochemistry*.

[15] Piao Y., Hung S. S.-C., Lim S. Y., Wong R. C.-B., Ko M. S. H. (2014). Efficient generation of integration-free human induced pluripotent stem cells from keratinocytes by simple transfection of episomal vectors. *Stem Cells Translational Medicine*.

[16] Okita K., Matsumura Y., Sato Y. (2011). A more efficient method to generate integration-free human iPS cells. *Nature Methods*.

[17] Lim S. Y., Sivakumaran P., Crombie D. E., Dusting G. J., Pébay A., Dilley R. J. (2013). Trichostatin A enhances differentiation of human induced pluripotent stem cells to cardiogenic cells for cardiac tissue engineering. *Stem Cells Translational Medicine*.

[18] Peters N. S., Severs N. J., Rothery S. M., Lincoln C., Yacoub M. H., Green C. R. (1994). Spatiotemporal relation between gap junctions and fascia adherens junctions during postnatal development of human ventricular myocardium. *Circulation*.

[19] Boulting G. L., Kiskinis E., Croft G. F. (2011). A functionally characterized test set of human induced pluripotent stem cells. *Nature Biotechnology*.

[20] Fijnvandraat A. C., Lekanne Deprez R. H., Moorman A. F. M. (2003). Development of heart muscle-cell diversity: a help or a hindrance for phenotyping embryonic stem cell-derived cardiomyocytes. *Cardiovascular Research*.

[21] Rajala K., Pekkanen-Mattila M., Aalto-Setälä K. (2011). Cardiac differentiation of pluripotent stem cells. *Stem Cells International*.

[22] Harding S. E., Ali N. N., Brito-Martins M., Gorelik J. (2007). The human embryonic stem cell-derived cardiomyocyte as a pharmacological model. *Pharmacology & Therapeutics*.

[23] van den Heuvel N. H. L., van Veen T. A. B., Lim B., Jonsson M. K. B. (2014). Lessons from the heart: mirroring electrophysiological characteristics during cardiac development to in vitro differentiation of stem cell derived cardiomyocytes. *Journal of Molecular and Cellular Cardiology*.

[24] Yang X., Pabon L., Murry C. E. (2014). Engineering adolescence: maturation of human pluripotent stem cell-derived cardiomyocytes. *Circulation Research*.

